# Gut microbiome composition reveals the distinctiveness between the Bengali people and the Indigenous ethnicities in Bangladesh

**DOI:** 10.1038/s42003-024-06191-9

**Published:** 2024-04-25

**Authors:** Ishtiaque Ahammad, Arittra Bhattacharjee, Zeshan Mahmud Chowdhury, Anisur Rahman, Mohammad Uzzal Hossain, Gourab Dewan, Shiny Talukder, Keshob Chandra Das, Chaman Ara Keya, Md Salimullah

**Affiliations:** 1 Bioinformatics Division, National Institute of Biotechnology, Ganakbari, Ashulia, Savar, Dhaka 1349 Bangladesh; 2 Rangamati Medical College, Hospital Road, Rangamati-4500 Rangamati, Bangladesh; 3 Molecular Biotechnology Division, National Institute of Biotechnology, Ganakbari, Ashulia, Savar, Dhaka 1349 Bangladesh; 4 Department of Biochemistry and Microbiology, North South University, Bashundhara, Dhaka 1229 Bangladesh

**Keywords:** Metagenomics, Genetics research, Microbiology

## Abstract

Ethnicity has a significant role in shaping the composition of the gut microbiome, which has implications in human physiology. This study intends to investigate the gut microbiome of Bengali people as well as several indigenous ethnicities (Chakma, Marma, Khyang, and Tripura) residing in the Chittagong Hill Tracts areas of Bangladesh. Following fecal sample collection from each population, part of the bacterial 16 s rRNA gene was amplified and sequenced using Illumina NovaSeq platform. Our findings indicated that Bangladeshi gut microbiota have a distinct diversity profile when compared to other countries. We also found out that Bangladeshi indigenous communities had a higher *Firmicutes* to *Bacteroidetes* ratio than the Bengali population. The investigation revealed an unclassified bacterium that was differentially abundant in Bengali samples while the genus *Alistipes* was found to be prevalent in Chakma samples. Further research on these bacteria might help understand diseases associated with these populations. Also, the current small sample-sized pilot study hindered the comprehensive understanding of the gut microbial diversity of the Bangladeshi population and its potential health implications. However, our study will help establish a basic understanding of the gut microbiome of the Bangladeshi population.

## Abstract (web summary)

Next-Generation Sequencing and analysis of 16 S rRNA gene from Bengali, Chakma, Marma, Khyang, and Tripura fecal samples revealed a distinct diversity profile of their gut microbiota compared to that of other countries.

## Introduction

The human gut microbiome which is composed of an enormous collection of microorganisms plays a crucial role in modulating various physiological processes, metabolic diseases, and immunological dysregulation. The composition of the gut microbiome is influenced by a multitude of factors, including diet, lifestyle, environment, genetics, and ethnicity^1^. They forge a symbiotic relationship with the host in the gastrointestinal tract, where they are monitored by the innate immune system using pattern recognition receptors such as toll-like receptors and Nucleotide-binding oligomerization domain-containing protein (NOD)-like receptors^2^. A number of these bacteria are involved in regulating host metabolism by producing metabolites such as folate, indoles, secondary bile acids, trimethylamine-N-oxide, neurotransmitters (e.g., serotonin, gamma amino butyric acid), and short-chain fatty acids^3^. Alteration in the host–symbiont relationships in the gut has been observed in diseases like obesity, type 2 diabetes mellitus, non-alcoholic liver disease, cardio-metabolic diseases, and so on^4^. Since different ethnic groups have been reported to have variations in genetics, food habits, lifestyle, their alterations can be greatly influenced by ethnicity. As a result, some ethnic groups are predisposed to certain metabolic diseases, while others are inclined to others^5^. For example, high-risk variants of *NOD2* are less prevalent in African Americans which makes their microbiome less prone to Crohn’s Disease^6,7^. Similarly, the *lactase* gene varies in different populations^8^. In the Japanese population higher abundance of *Bifidobacterium* has been observed due to their characteristic *LCT* genotypes^9,10^. Hence, gut microbiome studies on ethnic groups can be helpful in identifying prognostic microbial biomarkers for various diseases.

The genetic makeup, dietary patterns, and geographic regions shape the gut microbiome of various ethnicities in a distinct pattern^11–14^. Previously, a number of gut microbiome studies in South-East Asia have been conducted. Gut microbiomes of populations living in India and China which are geographically close to Bangladesh, showed an abundance of *Prevotella* and *Bacteroidetes* respectively^5^. In India, gut microbiomes from the Adi, Apatani, and Nyshi tribes of Arunachal Pradesh (a state in Northeastern India) demonstrated the predominance of *Firmicutes*, *Bacteroidates*, *Actinobacteria*, and *Proteobacteria*. On the other hand, Mongoloid and Proto-Australoid tribes of India were dominated by *Prevotella*^15,16^. However, studies from ethnic groups in Bangladesh are still not available. Bangladeshi ethnic groups have diverse dietary habits. Bengali food customs stem from their agrarian culture, which is defined by a profusion of rice, fish, and different vegetables. Dishes like biryani and kebabs are examples of the historical effects of Mughal and British dynasties on the cuisine. Seasonal fruit preparations, street snacks like shingara and samosas, and traditional cakes called “Pitha” are all common^17^. Bangladeshi tribal people use forest resources to eat diversely. They feed on ‘bans koral,’ bamboo shoots from *Melocanna baccifera* and *Bambusa tulda* during the rainy season. Fresh or dried wild plants commonly accompany staple grain recipes. Acidic leaves are often eaten as salad or chutney. Woody perennials including *Albizia procera*, wild mango, and *Daemonorops jenkinsianus* are vegetables for these ethnic groups. Lentinus, Shizophyllum, and Jew’s Ear mushrooms from decomposing wood are eaten. Wild banana inflorescences and leaf sheath soft cores are also edible. During food shortages, banana core cooked with rice or bran is crucial^18^.

Bangladesh is a country inhabited by many different ethnicities. Bengalis are the largest among them. Other major groups are Chakma, Marma, Khyang, and Tripura with distinguishable food habits and lifestyles. The Chittagong Hill Tracts in the southeastern parts of Bangladesh are home to the Chakma, Marma, Khyang, and Tripura people. Bengali people (Bengali language speakers) have Indian ancestry with Proto-Australoid, Caucasoid, and Mongoloid genetics^19–21^. On the other hand, Chakma, Marma, Khyang, and Tripura people (Tibeto-Burman language speakers) have predominance of Tibeto-Burman genetics. Tibeto-Burman populations originated from northwestern China and moved to the South. They have interbred with numerous southern tribes for the past 2600 years, creating specific genetic traits among southern Tibeto-Burman populations^22,23^. Hence, Tibeto-Burman speakers from Northeast India are more distinctive than the ones living in the Himalayas^23^. In Bangladesh, the Tibeto-Burman populations (e.g., Chakma, Marma, Khyang, Tripura etc.) have higher similarity with Northeast Indian Tibeto-Burman but they contain more mainland Indian ancestry^24^. In this study, we investigated the gut microbiomes of Bengali, Chakma, Marma, Khyang, and Tripura populations in order to determine whether Bangladeshi Tibeto-Burman populations differ from Bengali people in terms of their gut microbiomes.

## Methods

### Sample collection

Prior to sample collection, written informed consent was obtained from all participants. Ethical approval and necessary permits were obtained from the National Institute of Biotechnology Ethical Review Committee, Bangladesh (NIBERC2022-01). All ethical regulations relevant to human research participants were followed. Supplementary Data 1 contains detailed sample information regarding age, gender, and cohort.

All of the indigenous volunteers (Chakma, Marma, Khyang, and Tripura) in this study reside in the Rangamati district (22°37’60 N 92°12’0E) of the Chittagong Hill Tracts region. Bengali samples were collected from Dhaka Division (23.9536° N, 90.1495° E). Based on ethnicity, the participants were divided into five groups (Bengali, Chakma, Marma, Khyang, and Tripura). A total of 55 individuals were sampled, of which 13 were Bengali, 15 were Chakma, 10 were Khyang, 6 were Marma, and 11 were Tripura. Fecal samples of the participants were collected using sterile stool collection tubes. The samples were then transported to the National Institute of Biotechnology using Icebox and subsequently stored at −80 °C temperature. Fecal DNA extraction was executed using the PureLink™ Microbiome DNA Purification Kit (Catalog number: A29790). Specialized beads were used along with a combination of heat, chemical, and mechanical disruption to lyse the microorganisms efficiently. Precipitation with a proprietary cleaning buffer removed inhibitors. After that, the samples were placed in spin columns, and the DNA attached to the column was washed once before elution. DNA concentration and purity were estimated by Thermo Scientific NanoDrop 2000/2000c and then stored at −20 °C.

### Amplicon generation and library preparation for sequencing

Amplicons were generated using the 341 F (5’-CCTAYGGGRBGCASCAG-3’) and 806 R (5’-GGACTACNNGGGTATCTAAT-3’) primers that targeted the V3-V4 region of the bacterial 16 S rRNA gene. All PCR reactions were performed with 15 μL of Phusion® High-Fidelity PCR Master Mix (New England Biolabs), 0.2 μM of forward and reverse primers, and around 10 ng template DNA. For thermal cycling, initial denaturation at 98 °C for 1 min was followed by 30 cycles of denaturation at 98 °C for 10 s, annealing at 50 °C for 30 s, and elongation at 72 °C for 30 s. Final elongation was carried out for 5 min at 72 °C. TruSeq® DNA PCR-Free Sample Preparation Kit (Illumina, USA) was used as per the manufacturer’s protocol for sequencing library preparation, and index codes were appended. The quality of the library was evaluated by employing the Qubit@2.0 Fluorometer (Thermo Scientific) and the Agilent Bioanalyzer 2100 system. Finally, the library was sequenced on an Illumina NovaSeq platform, resulting in 250 bp paired-end reads.

### Data pre-processing and quality control

The paired end sequences were converted into QIIME2 format for data pre-processing, quality control, taxonomic assignment, differential abundance identification, functional analysis using QIIME2 platform (version 2021.4.0)^25^. More specifically, the data pre-processing of paired end sequences were performed using the DADA2 plug-in within QIIME2^26^. DADA2 filtered noisy reads, performed error correction in marginal sequences, removed chimeras and singletons, joined denoised paired-end reads, and also de-replicated the filtered reads. The features produced by DADA2 are denoted as amplicon sequence variants.

### Taxonomic assignment

For taxonomic assignment, a pre-trained classifier based on the Naive Bayes machine-learning model was implemented. This model was trained on 99% sequence similarity of Greengenes 13_8 data. This classifier was then deployed for taxonomy assignment of amplicon sequence variants.

### Diversity analysis

Several diversity metrics in QIIME2 require a rooted phylogenetic tree generated from the amplicon sequence variants of the sampled data. A reference-based fragment insertion method, using q2-fragment-insertion tool, was applied to construct the rooted tree for this purpose. Greengenes 13_8 data was used as a reference database in the q2-fragment-insertion tool^27,28^. The sequencing depth of the samples was 3525 to observe the richness. This phenomenon was checked with the alpha rarefaction curve generated by the q2-diversity tool. The microbiome within and between samples was calculated by the core-metric-phylogenetic method of the q2-diversity tool. This method computes several alpha (Observed features, Shanon diversity, Faith’s phylogenetic diversity, Pielou evenness) and beta (Jaccard distance, Bray–Curtis distance, unweighted UniFrac distance, and weighted UniFrac distance) diversity metrics altogether^29^. Based on each beta diversity metric, this command also performed principal coordinates analysis (PCoA)^30^. To visualize the PCoA plots for every beta diversity metric, EMPeror visualization tool was utilized to generate the figures^31^. Several statistical tests were conducted during diversity analysis such as Kruskal–Wallis H test, two-way ANOVA, paired *t*-test and PERMANOVA test^32–35^. The boxplot() function in R was used to draw boxplots based on alpha diversity values. This function follows the 1.5 IQR method for detecting outliers which are placed above and below the whiskers on the boxplots. Here, only one sample was identified as outlier (Sample ID: BBT19). The result including the outlier has been provided in Supplementary Fig. 1. After removal of the outlier, the number of samples used for analysis were reduced from 55 – 54.

### Differential abundance test

To classify the features that were differentially abundant across various sample groups the analysis of the composition of microbiomes (ANCOM) method was applied by the q2-composition tool^36^. This statistical framework was deployed at the genus level. The minimum sample size for each feature was set to 27 (half of the total samples) because ANCOM fails to manage false discovery rates at sample sizes <10 as well as to remove very lowly abundant features^37^. For linear discriminant analysis by the LEfSe, the feature table was collapsed at the genus level and the minimum sample size was chosen at 27^38^. This tool first performs the non-parametric Kruskal-Wallis (KW) sum-rank test to identify the features which had significant differential abundance across different metadata categories. Finally, LEfSe applies linear discriminant analysis to compute the effect size of each differentially abundant feature and plot the linear discriminant analysis score in the log10 scale. Result of both ANCOM and LEfSe analysis conducted for all samples including the previously mentioned outliers has been provided in Supplementary Fig. 2.

### Functional analysis

BURRITO, an interactive visualization web server (http://elbo-spice.cs.tau.ac.il/shiny/burrito/), was utilized to explore the taxa-function relationship within the samples of the study^39^. To acquire gene contents and functional annotations, this tool adopts the PICRUSt and KEGG Orthology databases, respectively^40,41^. At first, features with a sample size of <27 were filtered from the original feature table to remove very low abundant taxon. The q2-vsearch tool was employed for closed-reference clustering of retrieved features at 97% identity based on greengenes 97% OTU IDs as reference ^42,43^. Thus the acquired OTU table was then converted to the appropriate table format for BURRITO input.

### Comparative analysis with tropical and subtropical countries

Since Bangladesh is a tropical country, we compared the gut microbiome of Bangladeshi samples with several tropical and subtropical countries to see if there is any similarity between them. To do this we took sequence data from publicly available 16 s rRNA amplicon sequence data from the NCBI Sequence Read Archive and MG-RAST databases. Only healthy / control samples were taken from the selected datasets. Countries included in the comparative analysis were Australia, Egypt, India, Indonesia, Malaysia, Mexico, Thailand, and Vietnam. Information about the samples taken from NCBI and MG-RAST has been presented in Supplementary Data 2 and Supplementary Data 3 respectively. The samples downloaded from MG-RAST were processed using q2-deblur while the samples downloaded from NCBI were processed using q2-dada2^34,44^. This is because the samples were taken from different regions and were prepared and sequenced using different methods. Amplicon sequencing using the Illumina MiSeq technology was done on Indian samples, encompassing the V3-V4 region of bacterial 16 S rDNA. On the other hand, the V4 region of bacterial 16 S ribosomal RNA genes from Malawi, Amerindians, and the United States was amplified and sequenced using an Illumina HiSeq 2000. The V1-V3 region of the 16 S ribosomal RNA (rRNA) gene of the Mongolian samples was amplified and sequenced using pyrosequencing on a Roche GS FLX. The samples were merged with the qiime feature-table merge method. All the samples were rarefied to the same depth (3525).

These foreign samples (*n* = 181) were then compared with all Bangladeshi samples (*n* = 55) using the q2-diversity tool via Alpha diversity and Beta Diversity analysis. Afterwards, a phylogenetic rooted tree was generated by the sepp fragment insertion approach. This rooted phylogeny was used to create a Unweighted Pair Group Method with Arithmetic Mean tree based on the Unweighted UniFrac metric by beta-rarefaction command of the q2-diversity tool^45^.

### Reporting summary

Further information on research design is available in the Nature Portfolio Reporting Summary linked to this article.

## Results

### Bengali population possessed lower *Firmicutes* to *Bacteroidetes* ratio compared to Indigenous groups

Among the 19 classified phyla, *Firmicutes* and *Bacteroidetes* were the most prevalent (48% and 34% of the total) in the gut microbiome of all Bangladeshi populations. Other phyla with higher abundance were Proteobacteria (14%), Actinobacteria (3%), Tenericutes (0.5%), etc (Supplementary Fig. 3). Interestingly, 100% of taxa from the *Bacteroidetes* phylum belonged to the *Bacteroidia* class. On the other hand, considering *Firmicutes* phylum, 87% of features are *Clostridia* class, 11% *Bacilli*, and 2% are *Erysipelotrichi* (Supplementary Fig. 4). In Supplementary Data 4, the taxonomic classification of each feature id is documented, along with the confidence value.

**Fig. 1:**
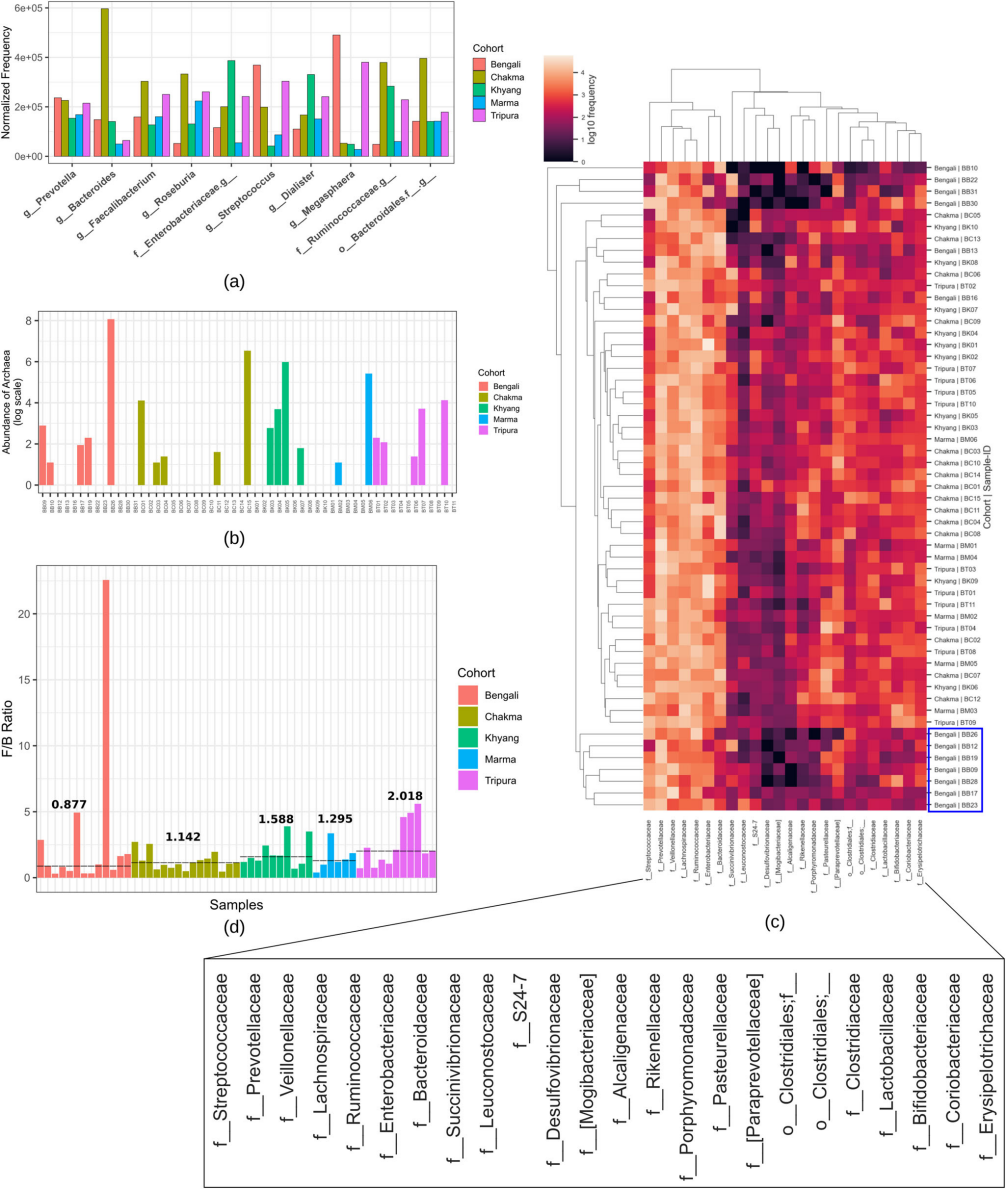
The abundance of the top prevailing bacterial genera, archaeal features, core features, and *Firmicutes* to *Bacteroidetes* ratio in various Bangladeshi cohorts. **a** The bar plot represents the normalized frequency of the top genera among different cohorts (**b**) Several archaeal species appeared to be present in different samples from each cohort. **c** The frequency of core features resulted in two primary clusters, with somewhat varied abundances in some Bengali samples. **d** The median ratio of *Firmicutes* to *Bacteroidetes*.

The normalized abundance of the top ten genera across different cohorts is represented in cpm (counts per million) (Fig. 1a). The *Prevotella* genus abundance was relatively similar in all cohorts. Nevertheless, the prevalence of the *Bacteroides* genus was drastically higher in the Chakma population and very low in Marma and Tripura samples. Moreover, Chakma tribal group also contained *Faecalibacterium*, *Roseburia*, and uncharacterized genera from the *Ruminococcaceae* family and *Bacteroidales* order in a relatively higher amount. On the other hand, *Streptococcus* and *Megasphaera* genera were highly abundant in the Bengali and Tripura populations and the Marma cohort contained these genera in relatively lower quantities. The Khyang group contained the genus *Dialister* and uncharacterized genera from *Enterobacteriaceae* and *Ruminococcaceae* families. About 0.05% amplicon sequence variants were classified as archaea of the *Methanobacteriaceae* family, of which only 0.1% were from the *Methanosphaera* genus, and the rest of the features belonged to the *Methanobrevibacter* genus (Fig. 1b). Twenty-four families of features were found as core features and the frequency heatmap of these features revealed that there were two main clusters, one with higher frequency and another with lower frequency (Fig. 1c). The *Streptococcaceae*, *Prevotellaceae*, *Veillonellaceae*, *Lachnospiraceae*, *Ruminococcaceae*, *Enterobacteriaceae*, and *Bacteroidaceae* families were in the higher frequency cluster. Most of the samples from the Bengali population clustered together and contained these higher frequency microbial families in relatively lower amounts. The median value of the *Firmicutes* to *Bacteroidetes* ratio was highest (2.018) in the case of Tripura samples, while the Bengali population had the lowest (0.877) median ratio (Fig. 1d). All other cohorts (Chakma, Khyang, Tripura) had a median *Firmicutes* to *Bacteroidetes* ratio of more than one.

### The Bengali population showed inferior microbiome richness than other

#### Ethnic cohorts

**Fig. 2:**
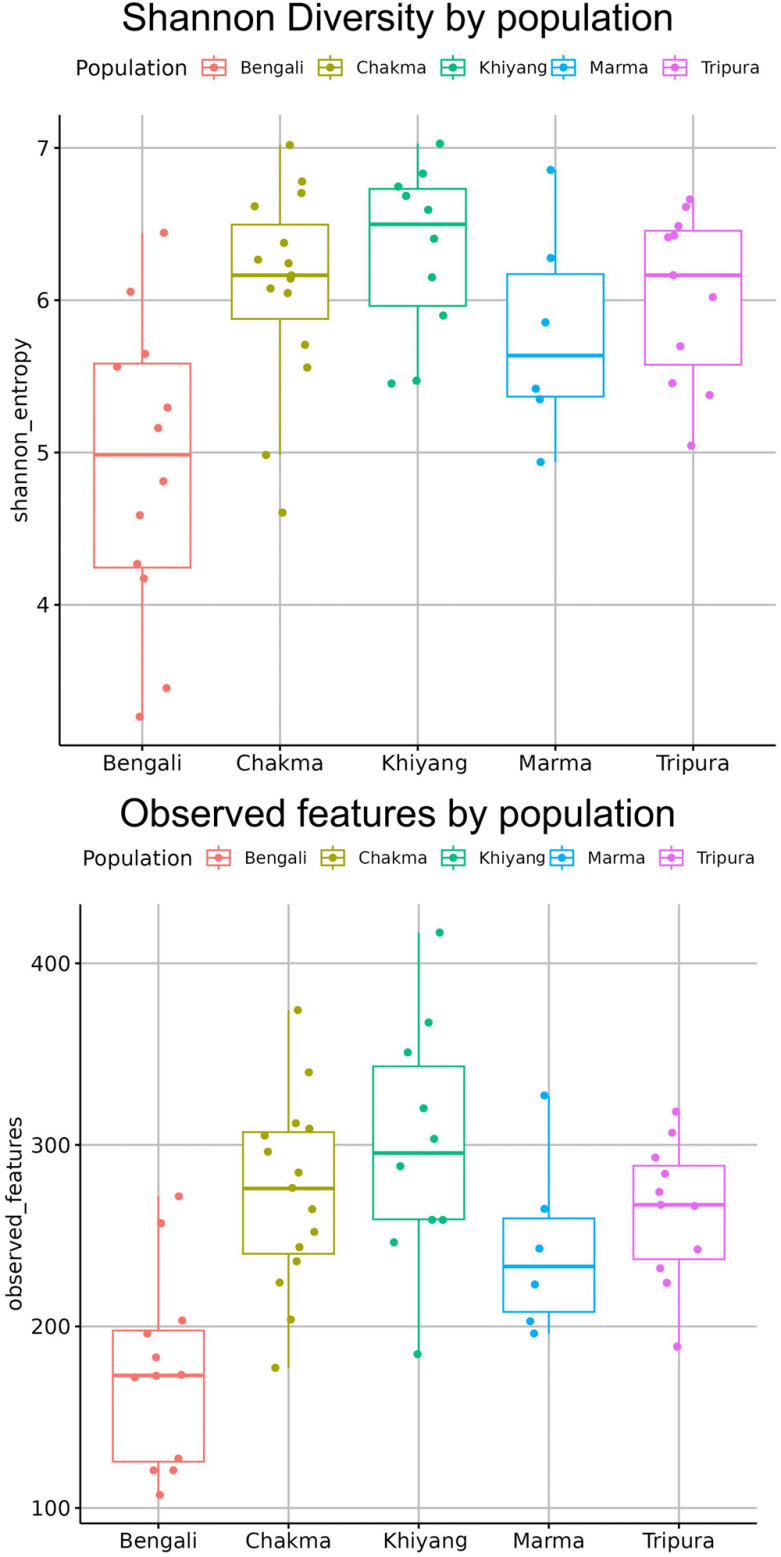
Distribution of alpha diversity in terms of Shannon diversity and observed features among various Bangladeshi ethnic cohorts. The Kruskal–Wallis test for all groups had a *p-*value of 0.0033. Chakma, Marma, Khyang and Tripura populations had higher species richness than the Bengalis (*p*-value = 0.0003) (*n* = 54 biologically independent samples).

**Fig. 3:**
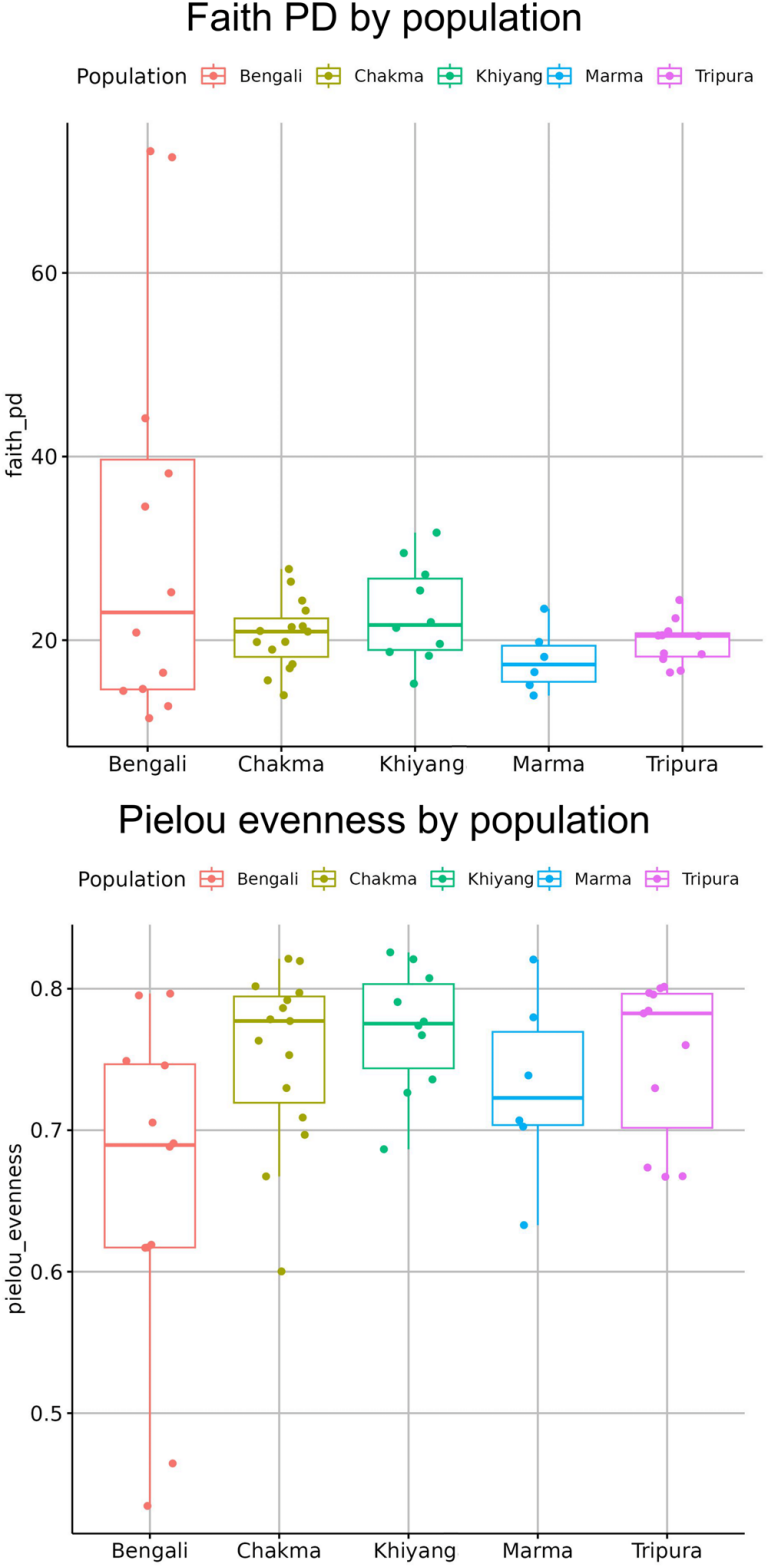
Distribution of alpha diversity in terms of Faith phylogenetic diversity and Pielou evenness among various Bangladeshi ethnic cohorts. The difference in Faith pd score was statistically insignificant (*p-*value = 0.3816). There was no significant difference in Pielou evenness profile between indigenous populations (*p-*value = 0.0631) (*n* = 54 biologically independent samples).

Alpha diversity depicts the diversity within the sample. Alpha diversity is measured via Shannon diversity, Observed features, Faith pd and Pielou evenness parameters. Shannon diversity calculates both the number of species in a community and their relative abundance. The median of Shannon diversity was relatively lower in the Bengali group compared to others (Fig. 2). For ethnicity based cohorts, Kruskal-Wallis test for all groups had a *p-*value of 0.0033 (Supplementary Data 5). Observed features counted the number of distinct features present in the cohorts (Fig. 2). Chakma, Marma, Khyang and Tripura populations had higher species richness than the Bengalis (*p-*value = 0.0003) (Supplementary Data 6). Faith pd incorporated information of the evolutionary relationships between different bacterial species. Bengali samples had lower Fatih *p*-values than indigenous populations (Fig. 3). However, the difference in Faith pd score was statistically insignificant (*p*-value = 0.3816) (Supplementary Data 7). Pielou evenness measured how evenly different species’ abundances were distributed within a community (Fig. 3). There was no significant difference in Pielou evenness profile between indigenous populations (*p-*value = 0.0631) (Supplementary Data 8).

#### Presence and absence of various species Made Bengali microbiome distinct

**Fig. 4:**
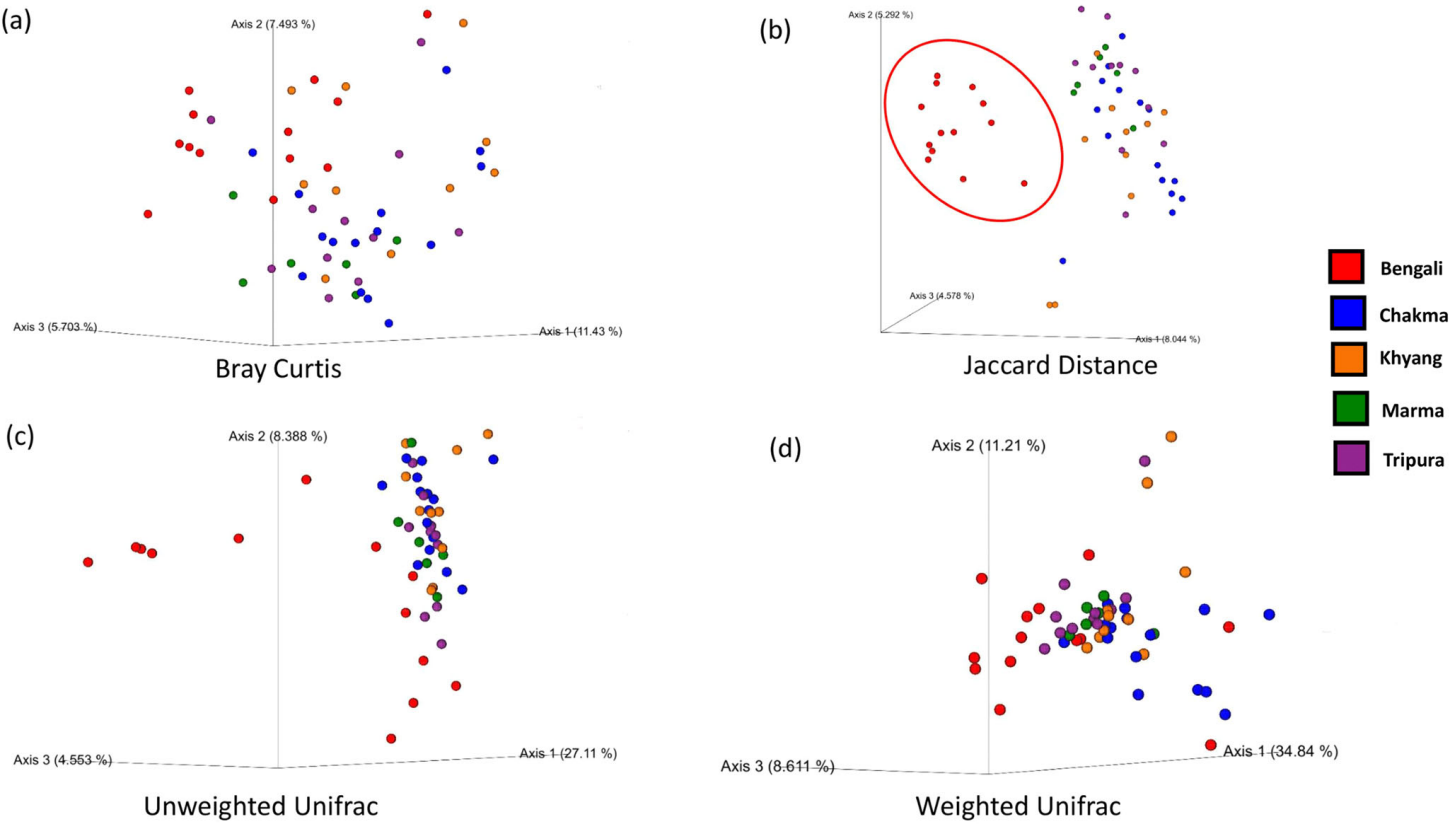
Beta diversity clustering between various Bangladeshi ethnic cohorts. The diversity was measured using (**a**) Bray–Curtis matrix (**b**) Jaccard distance matrix (**c**) Unweighted unifrac distance (**d**) Unweighted unifrac distance.

The PCoA plot revealed that the Bengali cohort was distinct from others in terms of Jaccard distance based on ethnicity (Fig. 4b) Jaccard distance is a binary distance matrix that only considers whether a taxon is present or not.

In other metrics, such as Bray–Curtis, weighted and unweighted UniFrac distances calculate presence and relative abundances of microbial taxa but no separated clusters were observed there (Figs. 4a, c, and d).

#### *Alistipes* genus is differentially abundant in Chakma population

**Fig. 5:**
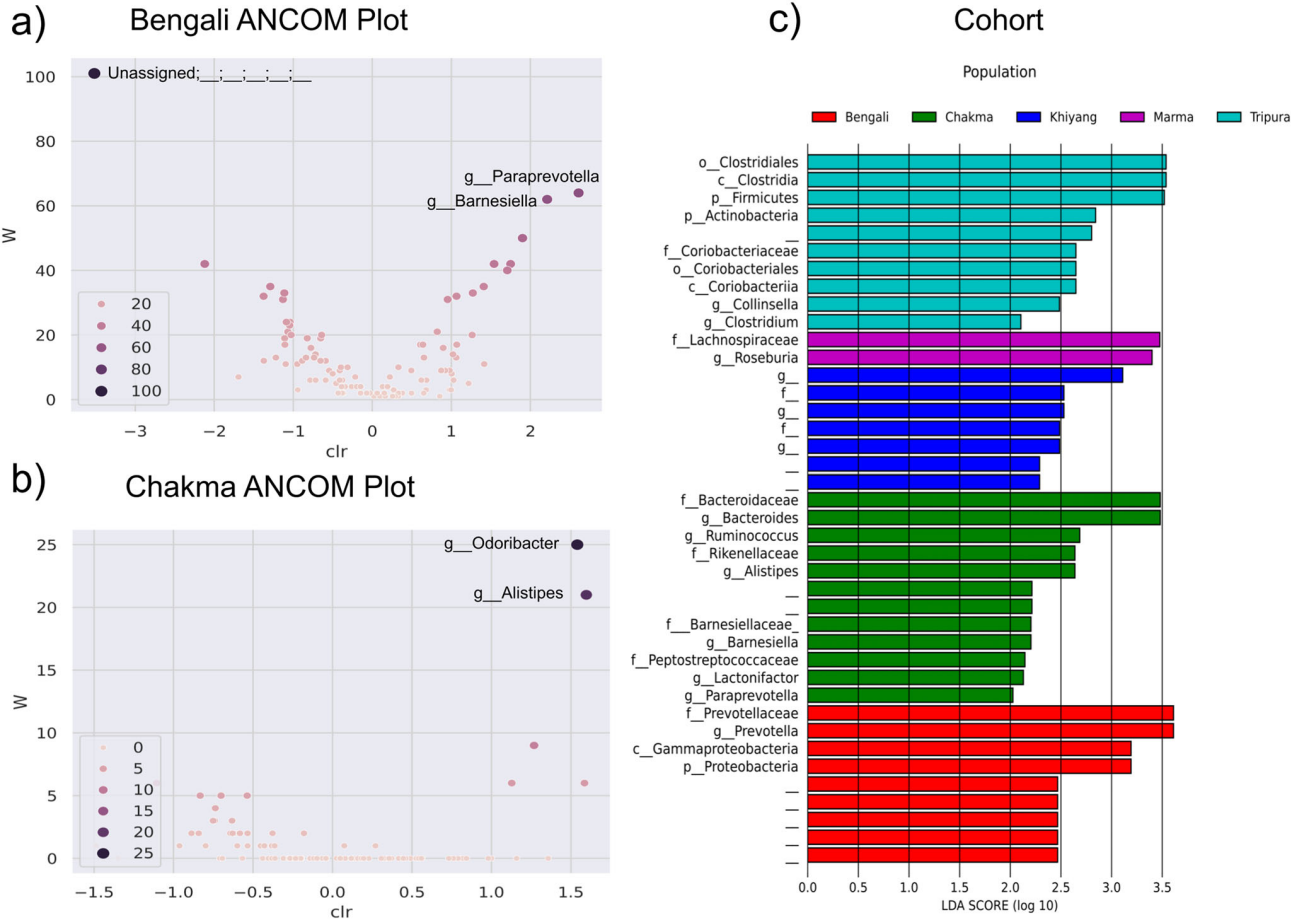
Differential abundance of gut microbes of various ethnic groups in Bangladesh. **a** ANCOM analysis of Bengali people (**b**) ANCOM analysis of Chakma people (**c**) LEfSe analysis of Bengali, Chakma, Marma, Khyang, and Tripura people (*n* = 54 biologically independent samples).

ANCOM analysis revealed the microbial community compositions between the various Bangladeshi ethnic groups. It used a log-ratio test to compare the relative abundance using non-parametric methods. Here, only the Bengali vs. Non-Bengali and the Chakma vs. Non-Chakma comparisons demonstrated differentially abundant taxa between the groups. In terms of ANCOM, the other groups (Marma, Khyang, and Tripura) did not reveal any differentially abundant bacteria. In the Bengali group, an unclassified bacteria showed a negative centered log ratio (clr) with higher W score (Fig. 5a). Whereas, the Chakma population depicted abundance for *Alistipes* and *Odoribacter* (Fig. 5b). Another tool, LEfSe implemented statistical methods to identify microbial features that were differentially abundant. LEfSe unveiled differentially abundant genus *Paraprevotella*, *Lactonifactor, Barnesiella, Bacteroides*, *Alistipes*, and *Ruminococcus* within the Chakma population (Fig. 5c). The results from ANCOM and LEfSe analysis complemented each other as they both unveiled that the genus *Alistipes* is differentially abundant in the Chakma population.

#### Bengali gut microbiome contributed to markedly diverse biological pathways

**Fig. 6:**
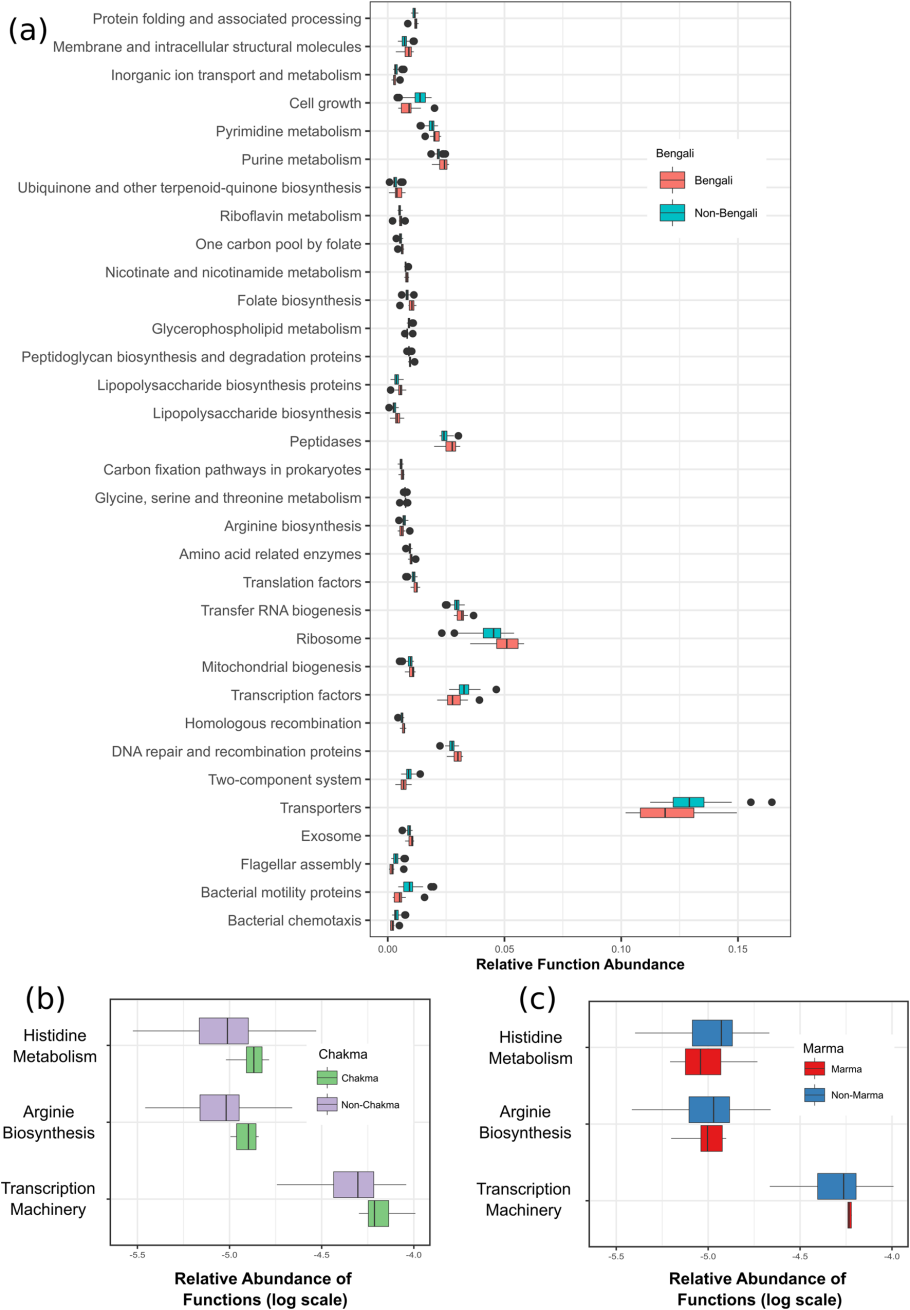
Functional analysis of enriched pathways in Bengali, Chakma, and Marma population. **a** Bengali (**b**) Chakma, and (**c**) Marma groups showed differential enrichment of function.

Relationships between microbiome composition and its effect on different biological functions were also explored in this study. Among all metadata categories, only the Bengali, Chakma, and Marma groups showed differential enrichment of function based on their gut microbiome composition (Fig. 6). All differentially abundant functions along with related contributing taxa and BH (Benjamini and Hochberg) FDR-adjusted *p*-values are tabulated in Supplementary Data 9. Thirty-three pathways were differentially abundant in Bengali samples. Several of them were highly enriched in Bengali samples such as peptidases, Ribosomes, Purine, and Pyrimidine metabolism (Fig. 6a). The genus *Bacteroides*, *Blautia*, *Collinsella*, *Coprococcus*, *Dorea*, *Parabacteroides*, SMB53, and *Slackia* were the top significantly contributing taxa in the enrichment of these functions. Chakma samples seemed to be enriched with histidine metabolism, arginine biosynthesis, and transcription machinery (Fig. 6b). But, in the case of the Marma samples histidine metabolism, arginine biosynthesis functions were in relatively lower abundance than other Non-Marma samples, and transcription machinery function was in higher frequency (Fig. 6c). For both categories, the genus *Bacteroides*, *Blautia*, and *Parabacteroides* were the most contributing taxa for function abundance. Detailed statistics for significant pathways and taxa contributions for each pathway are documented in Supplementary Data 10.

#### Bangladeshi gut microbiome has a distinct composition compared to tropical and subtropical populations

**Fig. 7:**
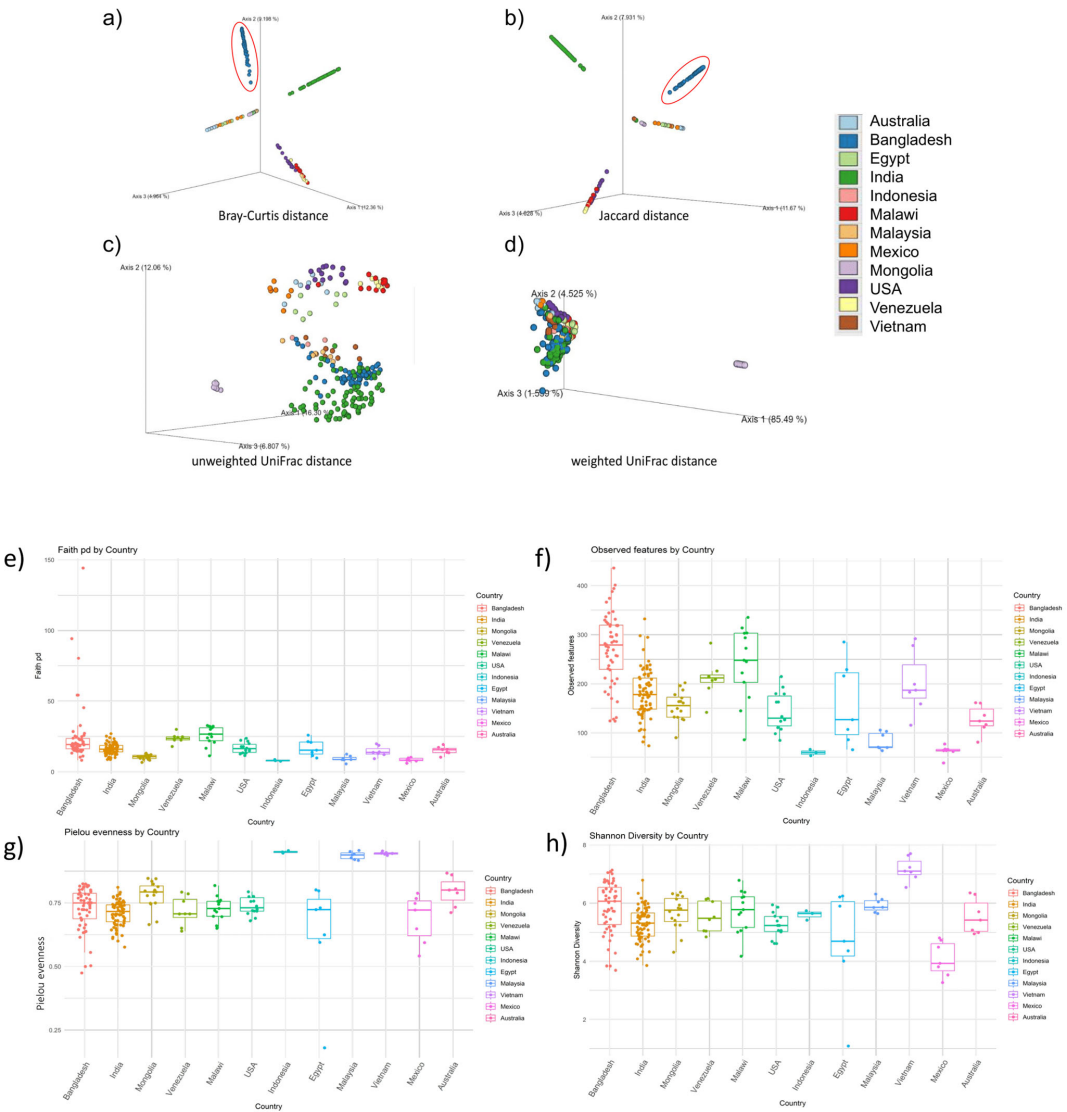
Comparative diversity analysis between Bangladesh and several other tropical and subtropical countries. **a** Bray–Curtis matrix (Red circle encircles Bangladeshi samples) (**b**) Jaccard distance matrix (Red circle encircles Bangladeshi samples) (**c**) Unweighted unifrac distance (**d**) Unweighted unifrac distance (**e**) Faith phylogenetic diversity (*p-*value = 2.3238) (**f**) Observed features (*p*-value = 1.6253) (**g**) Pielou evenness (*p*-value = 9.3489) (**h**) Shannon diversity (*p-*value = 6.0806) (*n* = 55 biologically independent samples).

**Fig. 8:**
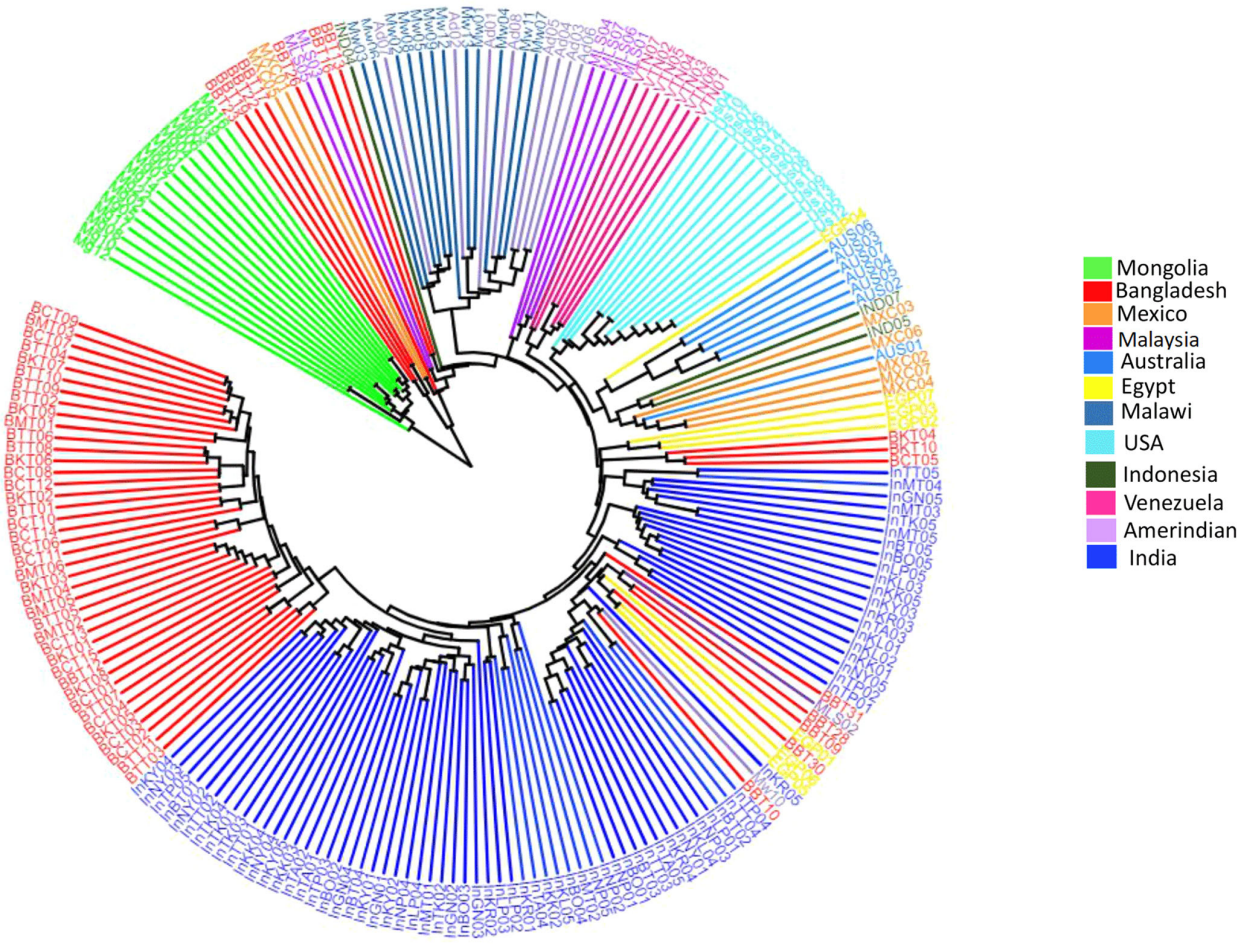
Unweighted pair group method with arithmetic mean based gut microbiome clustering of Bangladeshi and several other tropical and subtropical countries. Majority of the Bangladeshi indigenous samples clustered with Indian indigenous groups.

When compared with the gut microbiome of several other countries, the Bangladeshi samples formed a vividly distinct cluster based on bacterial composition and abundance (Fig. 7a, b). Cluster for Bray–Curtis and Jaccard distance indicates that bacterial composition and their abundances are unique for Bangladeshi populations. Their species richness was higher than other countries (Fig. 7f). The unweighted UniFrac, which incorporates phylogenetic information, demonstrated that most of the samples from the Bangladeshi population clustered closer to the Indian samples (Fig. 7c). All numerical source data of Fig. 7 can be found in Supplementary Data 11a–h. Furthermore, the Unweighted Pair Group Method with Arithmetic Mean based tree showed that most of the indigenous samples of Bangladesh have clustered with different Indian indigenous groups while the Bangladeshi samples as a whole were scattered across different phylogenetic clades (Fig. 8).

## Discussion

The human gut microbiome is an integral part of human growth and development and it is highly influenced by ethnicity^14,46^. Diverse ethnic populations reside in Bangladesh. The majority of the population is Bengali whereas large indigenous communities such as the Chakma, Marma, Tripura, and Khyang live in Chittagong Hill Tracts region of the country. The eating habits of these communities substantially differ from Bengali people. Their gut microbiomes, which may have an impact on important public health issues, can be influenced by their genetic make-up, geographic location, and lifestyle. In this study, we conducted a pilot survey to shed light of these areas.

Here, we have performed a 16 s rRNA gene amplicon sequencing to identify the gut microbiome composition. To execute this, we have collected fecal samples from Bengali, Chakma, Marma, Khyang, and Tripura populations. After collecting the microbial DNA from fecal samples, parts of the 16 S rRNA genes of the bacterial species were amplified. The amplified products were then sequenced. The sequence data was then processed and analyzed to draw interpretations. After all the analysis, we found that at the phylum level, Bengali population has lower *Firmicutes* to *Bacteroidetes* ratio compared to indigenous groups. Among *Firmicutes* phylum, *Dialister* and *Faecalibacterium* are highly abundant in Bangladeshi microbiomes (Fig. 1a). On the other hand, *Prevotella*, a member of the *Bacteroidetes* phylum, was distributed evenly across each cohort. People from the Indian subcontinent usually have *Prevotella* abundance in their gut^47^. Here, we have explored the alpha and beta diversity of the gut microbiome. Alpha diversity depicted within-group diversity and beta diversity showed different diversity ratios between the groups. The alpha diversity of the Bengali population was lower compared to others according to the Shannon diversity and species richness (Observed features) (Fig. 2). The overall species evenness was enriched for indigenous populations (Fig. 3). Based on the phylogenetic diversity of the gut microbes, the Bengali and the people of the Chittagong Hill Tracts were found not to be very distant. However, our findings indicate that the overall gut microbial population and abundance is unique for Bangladesh when compared with international datasets. However, most of the indigenous samples from Bangladesh (Chakma, Marma, Khyang, Tripura) clustered separately with a common origin with the Indian indigenous population whereas Bengali samples were spread across various clades (Fig. 8). Some bacterial species were found to be abundant in indigenous groups that were not enriched in Bengali samples or vice versa. To identify this differential abundance, we employed several statistical approaches using LEfSe and ANCOM. Both of these tools identified an unclassified bacteria, which can be studied further, that is significantly abundant in Bengali samples whereas Chakma samples had enriched levels of *Alistipes* species. *Alistipes* is a newly discovered genus under *Bacteroidetes* phylum.

The gut microbial pathways play a critical role in health and diseases^48^. In Bengali samples DNA repair system, pyrimidine and purine metabolism, lipopolysaccharide (LPS) biosynthesis, peptidases, ribosome, etc functions were enriched. On the other hand, Chakma samples were enriched with histidine metabolism and arginine biosynthesis (Fig. 6b) whereas in the Marma group abundance of these pathways were lower (Fig. 6c).

Furthermore, Bangladeshi gut microbes possessed a distinctive diversity profile. Further research is needed to investigate the specific differences in responses and underlying mechanisms associated with distinct gut microbiota profiles.

### Limitations of the study

The primary limitation of the study is small sample size, which exhibits significant variability among individuals sampled. With a sample size of *n* = 55 divided into five ethnic groups, two genders, and three large age groups (20–40, 40–60, and 60–80 years), the representativeness for each combination is low. The gut microbiome (GM) is well-known to be highly influenced by factors such as sex, age, current health status and so on. In our study, the representativeness of each of these groups is limited. A higher sample size would certainly enhance the credibility of the differences between these groups and also help draw more meaningful conclusions. However, the primary goal of our study was to explore and characterize the gut microbiome of Bangladeshi population for the first time and thus establish the baseline data for further gut microbiome research in Bangladesh. During the course of the study, as a secondary goal, we also wanted to see if there is any difference in the gut microbiome of the various ethnicities. Since our main priority was to characterize the Bangladeshi population as a whole, the difference between various subgroups based on age, sex, and health status was given less importance.

We want to reiterate that the number of participants in the current study was not sufficient to provide a comprehensive representation of the overall gut microbial diversity within neither the Bangladeshi population as a whole nor the ethnicities studied (Bengali, Chakma, Marma, Khyang, and Tripura). As a result, the findings from this study may lack statistical power and robustness, leading to potential biases and limited generalizability of the results. Moreover, the small sample size may hinder the identification of subtle microbial variations that could be crucial in understanding complex interactions between the gut microbiota and various health conditions. Consequently, caution must be exercised when interpreting the results of this gut microbiome study due to its limited sample size. Further research with larger cohorts is absolutely necessary to draw more definitive and reliable conclusions about the role of gut microbiota in the Bangladeshi population.

In conclusion, the current study indicates that the indigenous gut microbiome was more diverse and distinct from the Bengali population. Our study will help establish the baseline data for the gut microbiome of the Bangladeshi population.

### Supplementary information

Supplementary Information

Description of Supplementary Materials

Supplementary Data 1-11

Reporting Summary

## Data Availability

All newly generated 16rRNA amplicon sequencing data used in this study can be freely accessed via NCBI BioProject number PRJNA876782. Source data have been included in the supplementary data files. All other data are available from the corresponding author on reasonable request.

## Code availability

Code can be accessed from the corresponding author on reasonable request. Versions of software used to process the current dataset: QIIME2 (version 2021.4.0).
