# Supplementary material for: Gut microbiome composition reveals the distinctiveness between the Bengali people and the Indigenous ethnicities in Bangladesh
Source: Commun Biol. 2024 Apr 25;7:500. doi: 10.1038/s42003-024-06191-9 (PMC11045797; doi:10.1038/s42003-024-06191-9)
Supplement: Supplementary file 1 — Supplementary Information [file 42003_2024_6191_MOESM1_ESM.pdf]

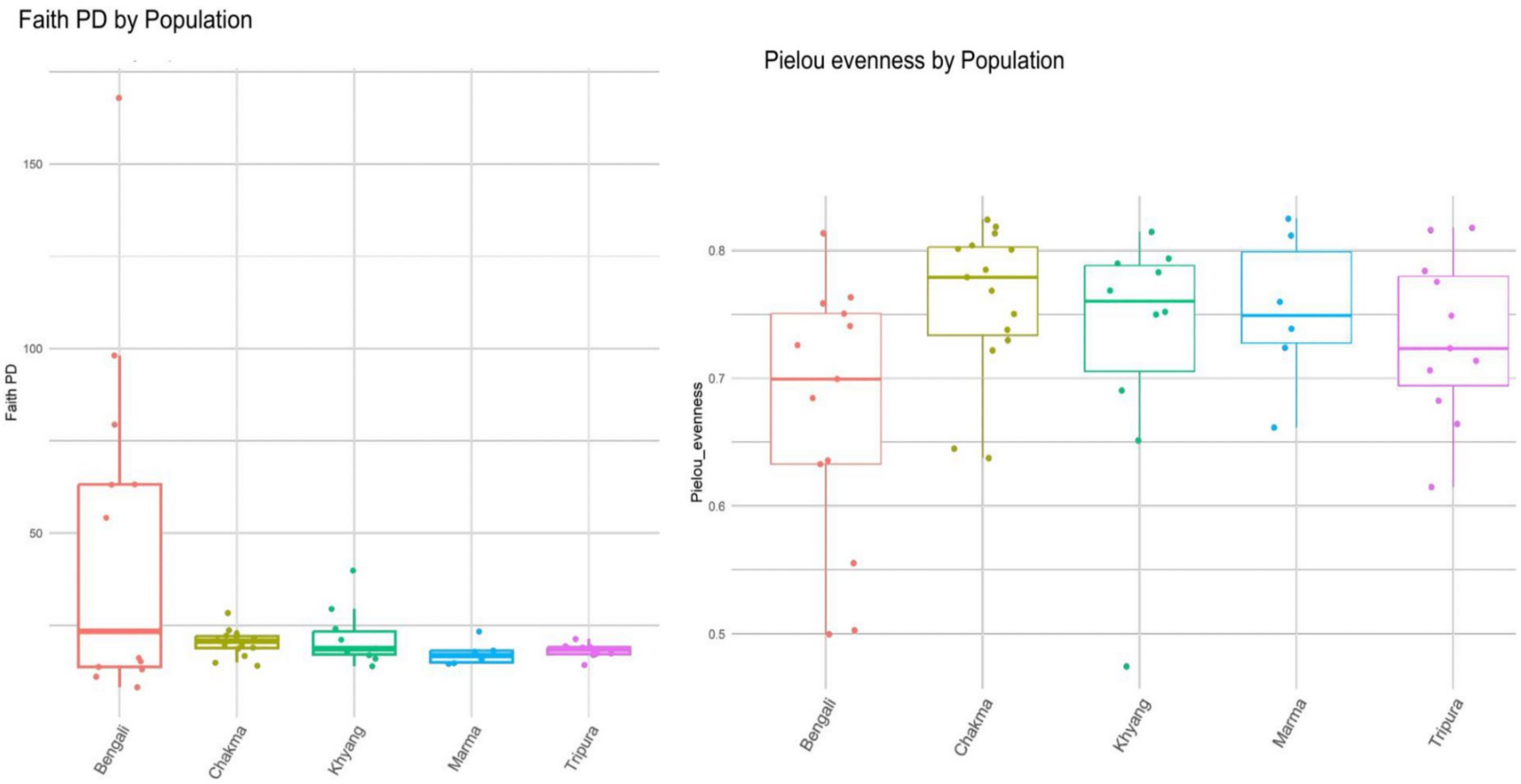

Supplementary Figure 1: Distribution of alpha diversity in terms of Faith phylogenetic diversity and Pielou evenness among various Bangladeshi ethnic cohorts.

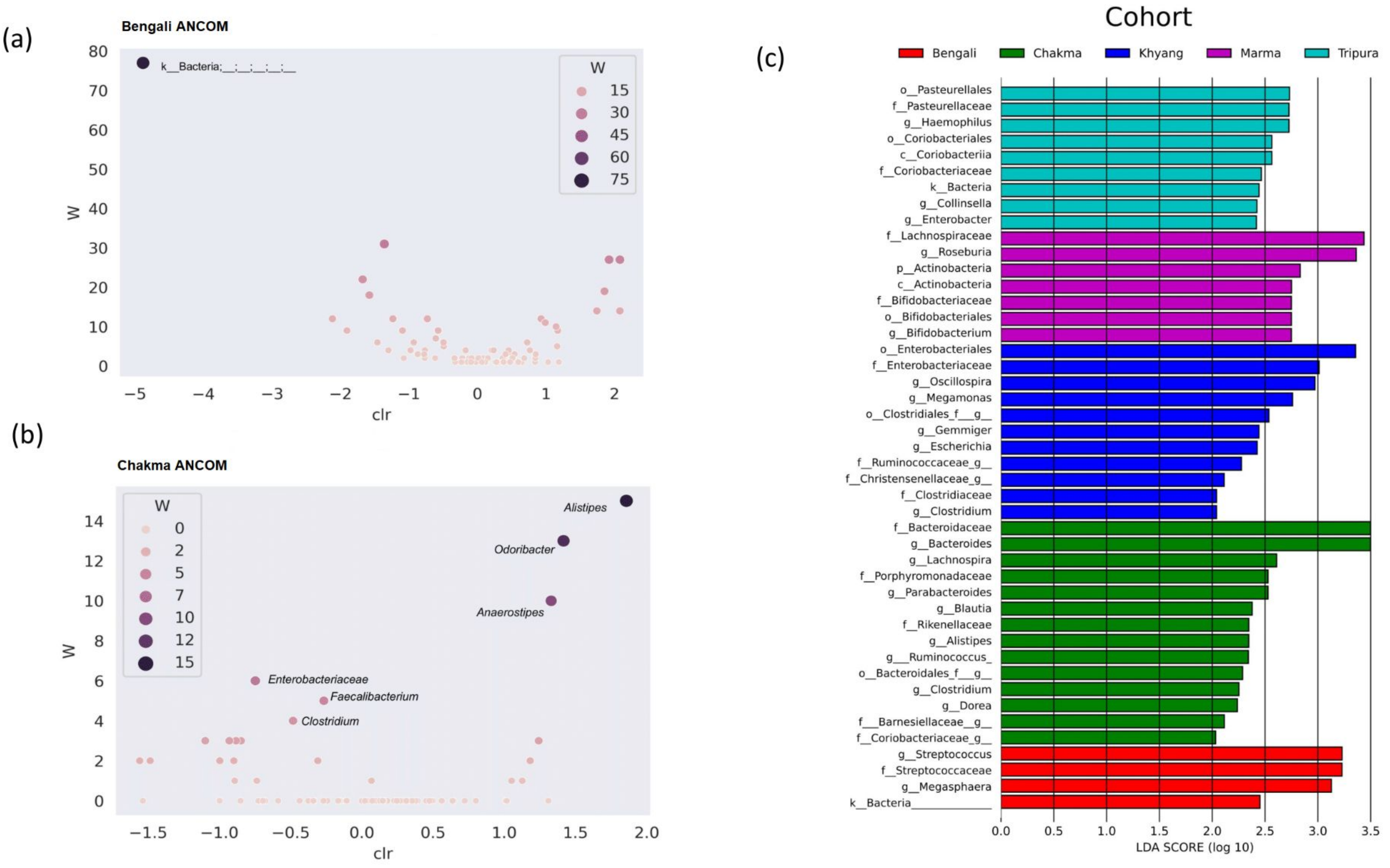

Supplementary Figure 2: Differential abundance of gut microbes of various ethnic groups in Bangladesh. (a) ANCOM analysis of Bengali people (b) ANCOM analysis of Chakma people (c) LEfSe analysis of Bengali, Chakma, Marma, Khyang, and Tripura people.

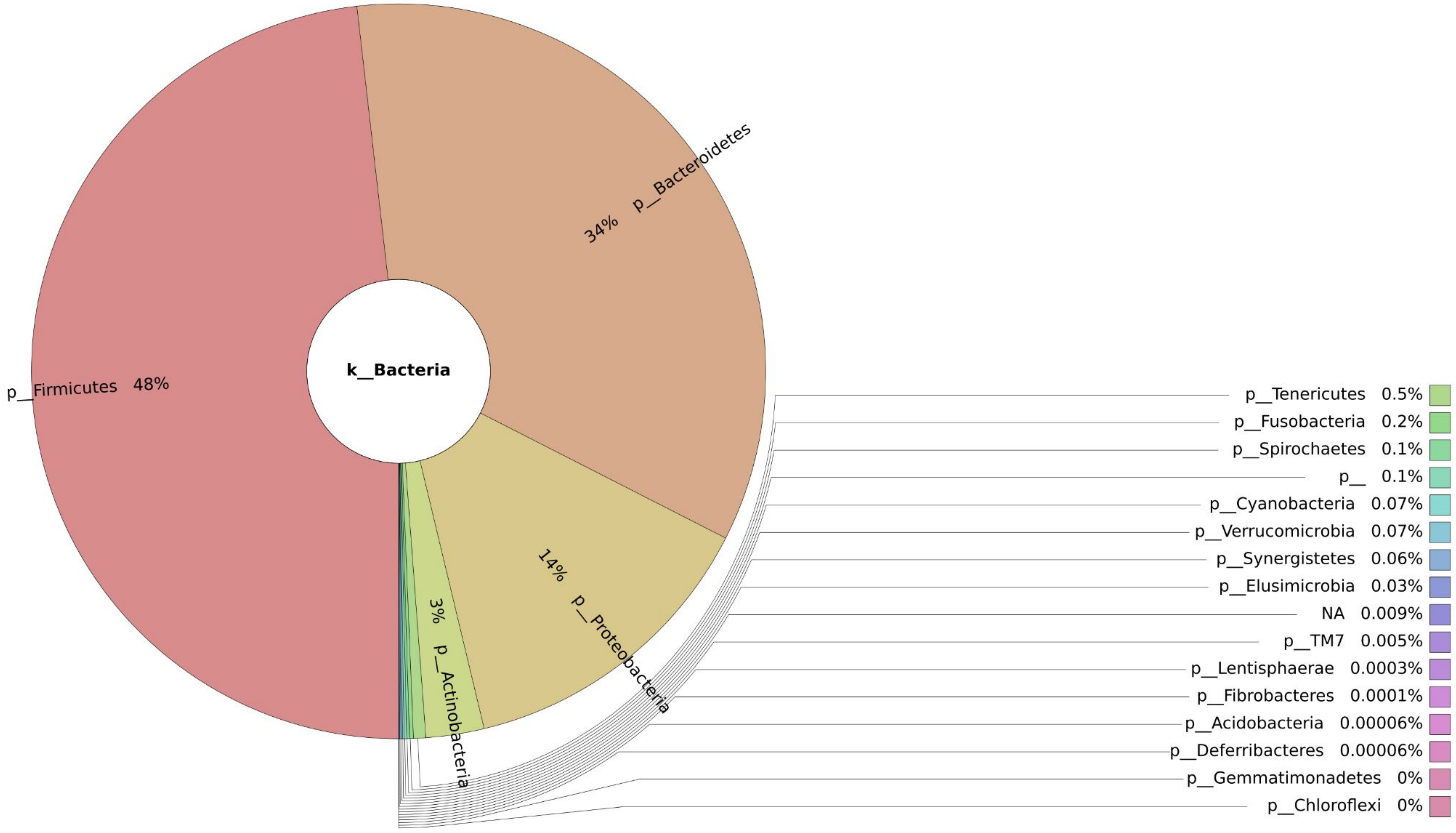

Supplementary Figure 3: Krona plot at phylum level. There were a total of 19 recognized phyla found in all 55 Bangladeshi samples. Firmicutes and Bacteroidetes were the most prevalent, accounting for 48% and 34% of the total, respectively. Moreover, the Bangladeshi population had a higher prevalence of the Proteobacteria phylum (14%) in their gut as well.

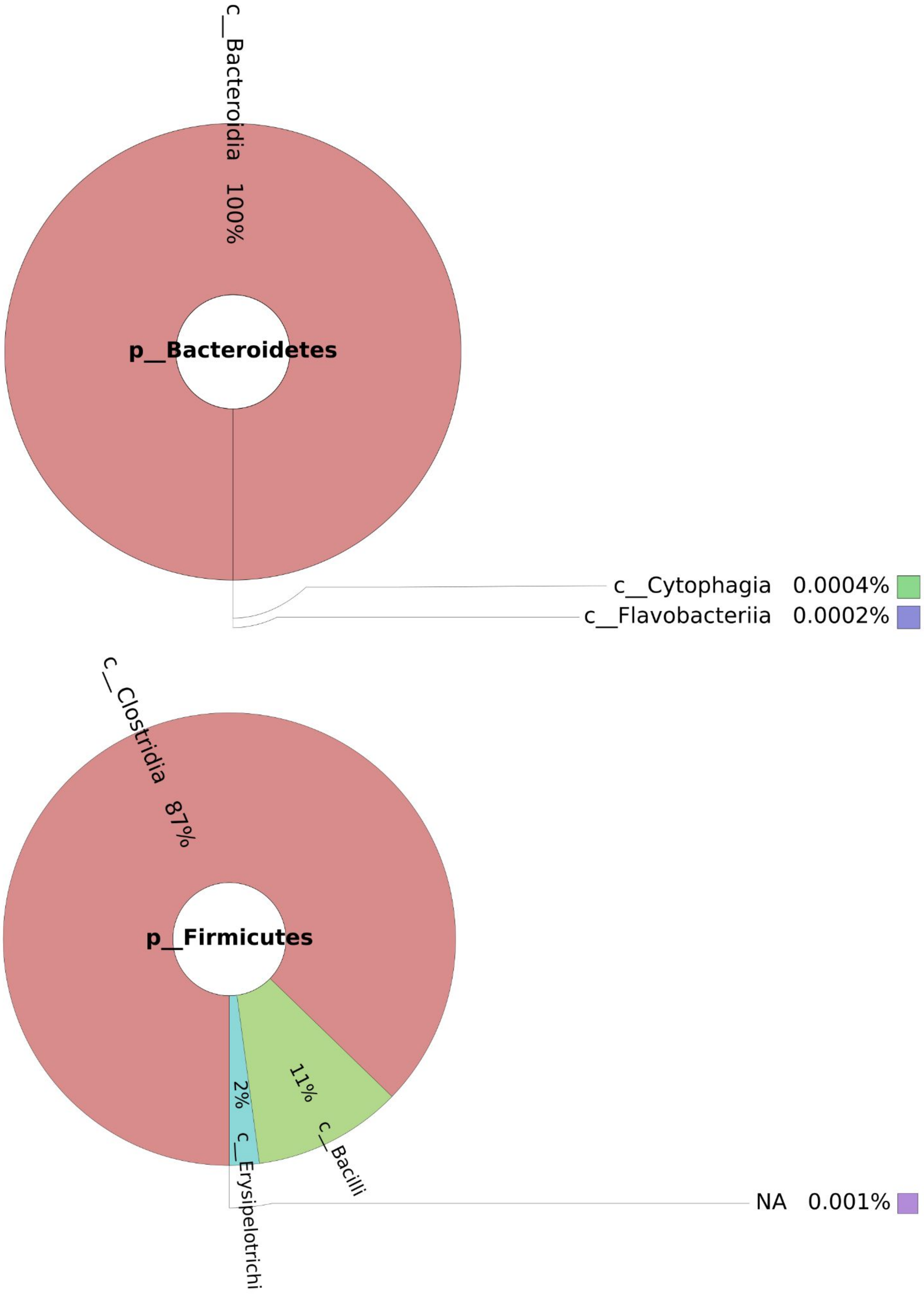

Supplementary Figure 4: Distribution of taxa within the phyla Firmicutes and Bacteroidetes.
