# Supplementary material for: Gut microbiome composition reveals the distinctiveness between the Bengali people and the Indigenous ethnicities in Bangladesh
Source: Commun Biol. 2024 Apr 25;7:500. doi: 10.1038/s42003-024-06191-9 (PMC11045797; doi:10.1038/s42003-024-06191-9)
Supplement: Supplementary file 2 — Description of Supplementary Materials [file 42003_2024_6191_MOESM2_ESM.docx]

1 Description of Additional Supplementary Files

2

1. **File name:** Supplementary Data 1
2. **Description:** Sample information for all Bangladeshi data generated in the current study.
3. **File name:** Supplementary Data 2
4. **Description:** Sample information for international data (Thailand, Indonesia, Egypt, Malaysia, Vietnam, Mexico, Australia) retrieved from NCBI.
5. **File name:** Supplementary Data 3
6. **Description:** Sample information for international data (India, Mongolia, Venezuela, Malawi, USA) retrieved from MG-RAST.
7. **File name:** Supplementary Data 4
8. **Description:** Taxonomic classification along with the confidence value for all Bangladeshi samples.
9. **File name:** Supplementary Data 5
10. **Description:** Shannon diversity for all Bangladeshi ethnic cohorts (Bengali, Chakma, Marma, Khyang, and Tripura).
11. **File name:** Supplementary Data 6
12. **Description:** Observed features for all Bangladeshi ethnic cohorts (Bengali, Chakma, Marma, Khyang, and Tripura).
13. **File name:** Supplementary Data 7
14. **Description:** Faith pd for all Bangladeshi ethnic cohorts (Bengali, Chakma, Marma, Khyang, and Tripura).
15. **File name:** Supplementary Data 8
16. **Description:** Pielou evenness or all Bangladeshi ethnic cohorts (Bengali, Chakma, Marma, Khyang, and Tripura).
17. **File name:** Supplementary Data 9
18. **Description:** Differentially abundant functions and related contributing taxa in Bengali samples.
19. **File name:** Supplementary Data 10
20. **Description:** Differentially abundant pathways and taxa contributions for each pathway in Bengali samples.
21. **File name:** Supplementary Data 11
22. **Description:** All numerical source data for (a) Bray Curtis matrix (b) Jaccard distance matrix (c) Unweighted unifrac distance (d) Weighted unifrac distance (e) Faith phylogenetic diversity (f) Observed features (g) Pielou evenness (h) Shannon diversity.
